# Association between breakfast frequency and physical activity and sedentary time: a cross-sectional study in children from 12 countries

**DOI:** 10.1186/s12889-019-6542-6

**Published:** 2019-02-21

**Authors:** Julia K. Zakrzewski-Fruer, Fiona B. Gillison, Peter T. Katzmarzyk, Emily F. Mire, Stephanie T. Broyles, Catherine M. Champagne, Jean-Philippe Chaput, Kara D. Denstel, Mikael Fogelholm, Gang Hu, Estelle V. Lambert, Carol Maher, José Maia, Tim Olds, Vincent Onywera, Olga L. Sarmiento, Mark S. Tremblay, Catrine Tudor-Locke, Martyn Standage, for the ISCOLE Research Group, Peter T. Katzmarzyk, Denise G. Lambert, Tiago Barreira, Stephanie Broyles, Ben Butitta, Catherine Champagne, Shannon Cocreham, Kara Denstel, Katy Drazba, Deirdre Harrington, William Johnson, Dione Milauskas, Emily Mire, Allison Tohme, Ruben Rodarte, Bobby Amoroso, John Luopa, Rebecca Neiberg, Scott Rushing, Timothy Olds, Carol Maher, Lucy Lewis, Katia Ferrar, Effie Georgiadis, Rebecca Stanley, Victor Keihan Rodrigues Matsudo, Sandra Matsudo, Timoteo Araujo, Luis Carlos de Oliveira, Leandro Rezende, Luis Fabiano, Diogo Bezerra, Gerson Ferrari, Mark S. Tremblay, Jean-Philippe Chaput, Priscilla Bélanger, Mike Borghese, Charles Boyer, Allana LeBlanc, Claire Francis, Geneviève Leduc, Pei Zhao Gang Hu, Chengming Diao, Wei Li, Weiqin Li, Enqing Liu, Gongshu Liu, Hongyan Liu, Jian Ma, Yijuan Qiao, Huiguang Tian, Yue Wang, Tao Zhang, Fuxia Zhang, Olga Sarmiento, Julio Acosta, Yalta Alvira, Maria Paula Diaz, Rocio Gamez, Maria Paula Garcia, Luis Guillermo Gómez, Lisseth Gonzalez, Silvia Gonzalez, Carlos Grijalba, Leidys Gutierrez, David Leal, Nicolas Lemus, Etelvina Mahecha, Maria Paula Mahecha, Rosalba Mahecha, Andrea Ramirez, Paola Rios, Andres Suarez, Camilo Triana, Mikael Fogelholm, Elli Hovi, Jemina Kivelä, Sari Räsänen, Sanna Roito, Taru Saloheimo, Leena Valta, Anura Kurpad, Rebecca Kuriyan, Deepa P. Lokesh, Michelle Stephanie D’Almeida, R. Annie Mattilda, Lygia Correa, D. Vijay, Vincent Onywera, Mark S. Tremblay, Lucy-Joy Wachira, Stella Muthuri, Jose Maia, Alessandra da Silva Borges, Sofia Oliveira Sá Cachada, Raquel Nichele de Chaves, Thayse Natacha Queiroz Ferreira Gomes, Sara Isabel Sampaio Pereira, Daniel Monteiro de Vilhena E Santos, Fernanda Karina dos Santos, Pedro Gil Rodrigues da Silva, Michele Caroline de Souza, Vicki Lambert, Matthew April, Monika Uys, Nirmala Naidoo, Nandi Synyanya, Madelaine Carstens, Martyn Standage, Sean Cumming, Clemens Drenowatz, Lydia Emm, Fiona Gillison, Julia Zakrzewski, Catrine Tudor-Locke, Ashley Braud, Sheletta Donatto, Corbin Lemon, Ana Jackson, Ashunti Pearson, Gina Pennington, Daniel Ragus, Ryan Roubion, John Schuna, Derek Wiltz, Alan Batterham, Jacqueline Kerr, Michael Pratt, Angelo Pietrobelli

**Affiliations:** 1 Institute for Sport and Physical Activity Research, University of Bedfordshire, Bedford, UK; 2 Department for Health, University of Bath, Bath, UK; 3 Pennington Biomedical Research Center, Baton Rouge, LA USA; 4 CHEO, Ottawa, Canada; 5 Department of Food and Nutrition, University of Helsinki, Helsinki, Finland; 6 UCT Research Centre for Health through Physical Activity, Lifestyle and Sport (HPALS), Division of Exercise Science and Sports Medicine, Faculty of Health Sciences, University of Cape Town, Cape Town, South Africa; 7 Alliance for Research In Exercise Nutrition and Activity (ARENA), School of Health Sciences, University of South Australia, Adelaide, Australia; 8 CIFI2D, Faculdade de Desporto, University of Porto, Porto, Portugal; 9 Department of Recreation Management and Exercise Science, Kenyatta University, Nairobi, Kenya; 10 School of Medicine Universidad de los Andes, Bogota, Colombia; 11 Department of Kinesiology, University of Massachusetts Amherst, Amherst, USA

**Keywords:** Exercise, Health, Fasting, International, Nutrition, Youth

## Abstract

**Background:**

Existing research has documented inconsistent findings for the associations among breakfast frequency, physical activity (PA), and sedentary time in children. The primary aim of this study was to examine the associations among breakfast frequency and objectively-measured PA and sedentary time in a sample of children from 12 countries representing a wide range of human development, economic development and inequality. The secondary aim was to examine interactions of these associations between study sites.

**Methods:**

This multinational, cross-sectional study included 6228 children aged 9–11 years from the 12 International Study of Childhood Obesity, Lifestyle and the Environment sites. Multilevel statistical models were used to examine associations between self-reported habitual breakfast frequency defined using three categories (breakfast consumed 0 to 2 days/week [rare], 3 to 5 days/week [occasional] or 6 to 7 days/week [frequent]) or two categories (breakfast consumed less than daily or daily) and accelerometry-derived PA and sedentary time during the morning (wake time to 1200 h) and afternoon (1200 h to bed time) with study site included as an interaction term. Model covariates included age, sex, highest parental education, body mass index z-score, and accelerometer waking wear time.

**Results:**

Participants averaged 60 (s.d. 25) min/day in moderate-to-vigorous PA (MVPA), 315 (s.d. 53) min/day in light PA and 513 (s.d. 69) min/day sedentary. Controlling for covariates, breakfast frequency was not significantly associated with total daily or afternoon PA and sedentary time. For the morning, frequent breakfast consumption was associated with a higher proportion of time in MVPA (0.3%), higher proportion of time in light PA (1.0%) and lower min/day and proportion of time sedentary (3.4 min/day and 1.3%) than rare breakfast consumption (all *p* ≤ 0.05). No significant associations were found when comparing occasional with rare or frequent breakfast consumption, or daily with less than daily breakfast consumption. Very few significant interactions with study site were found.

**Conclusions:**

In this multinational sample of children, frequent breakfast consumption was associated with higher MVPA and light PA time and lower sedentary time in the morning when compared with rare breakfast consumption, although the small magnitude of the associations may lack clinical relevance.

**Trial registration:**

The International Study of Childhood Obesity, Lifestyle and the Environment (ISCOLE) is registered at (Identifier NCT01722500).

**Electronic supplementary material:**

The online version of this article (10.1186/s12889-019-6542-6) contains supplementary material, which is available to authorized users.

## Background

Frequent breakfast consumption is associated with lower levels of obesity and lower chronic disease risk factors in children [1–3]. Yet, globally around one third of children do not consume breakfast daily [3, 4]. The question remains as to whether consuming breakfast regularly causes a reduction in obesity risk, through lower daily energy intakes or higher physical activity (PA), or whether breakfast consumption is an indicator of healthy lifestyle habits [5]. Nevertheless, total daily energy intake is not lower in children who consume breakfast frequently [6, 7], suggesting that PA levels may be higher among these children.

Although the majority of cross-sectional studies show that more frequent breakfast consumption is associated with higher self-reported PA in children [7–9], others report no such association [10, 11]. Of the few studies that have used an objective PA measure (e.g., accelerometry), equivocal findings have been reported. Indeed, more frequent breakfast consumption has been shown to be associated with higher PA in girls [12, 13] and in boys [14] in some studies, while in others, the relationship between physical activity and breakfast frequency was not supported in girls [14] or boys [12] or only occurred on weekends and not weekdays [15]. If they do exist, such associations may be strongest in the morning and weaken as the day progresses [12], as has been the case in randomised controlled trials with adults [16–18]. Yet, differences in the definition of ‘breakfast’ [19], PA assessment methods [20], and sociocultural differences in the countries within which the research has been conducted prevents direct comparisons between these studies and may explain some disparities in these findings. Moreover, findings from single-country studies may not apply across different regions of the world.

Few multi-national studies have examined the association between breakfast frequency and PA or sedentary time [21, 22]. From the limited work available, and much like the single-country research, inconsistent findings have been reported. In a sample of 11 to 15 year-old children from 41 countries across Europe, the U.S., Canada, and Israel, participants classified as ‘daily breakfast consumers’ reported that they participated in more moderate- to vigorous-intensity PA (MVPA) in 34 of the 41 countries and were less likely to watch more than two hours of television each day in 30 of the 41 countries compared with those consuming breakfast ‘less than daily’ [21]. In contrast, breakfast frequency was not associated with objectively measured or self-reported MVPA or accelerometer counts/min in adolescents from nine European countries [22]. Although 24-h recall breakfast consumption was related to measured sedentary time, no associations were found when breakfast patterns were assessed via questionnaire and when sedentary time was self-reported [22]. Yet, the degree of cross-country similarity or differences in the findings was not analyzed in these studies and the limited range of cultural and socioeconomic (SES) diversity in the study sites prevents global application of the findings [21, 22]. As we have previously shown the association between breakfast frequency and adiposity indicators differs in children from 12 countries spanning diverse cultural and SES backgrounds [3], these are important considerations.

Using data from the International Study of Childhood Obesity, Lifestyle, and the Environment (ISCOLE [23]), the primary aim of the present study was to examine the association of breakfast frequency with objectively-assessed PA and sedentary time in 9–11 year-old children. A secondary aim was to examine whether these associations differ between study sites varying diversely in terms of geographic region and human and SES development. We hypothesised that breakfast frequency would be positively associated with PA and negatively associated with sedentary time, particularly in the morning; further, the direction of these associations may be reversed in countries with lower levels of SES development.

## Methods

### Study design and participants

**Fig. 1:**
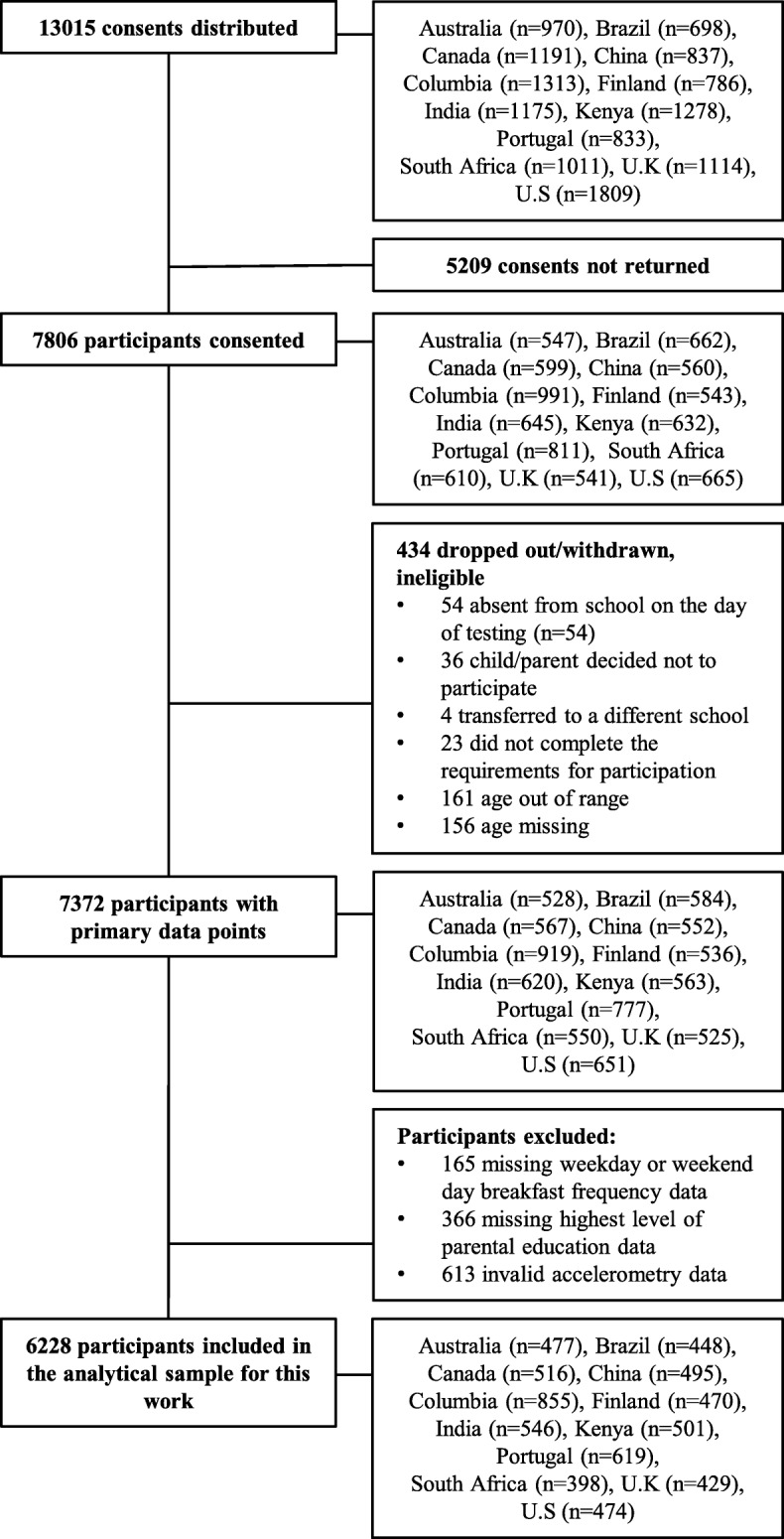
Participant flow chart

This cross-sectional, multi-national study was designed to determine the relationships between lifestyle behaviors and obesity in 12 study sites that represented a wide range of economic development (low to very high income), Human Development Index (0.509 in Kenya to 0.929 in Australia), and inequality (GINI coefficient; 26.9 in Finland to 63.1 in South Africa) [23]. The 12 study sites were located in Australia, Brazil, Canada, China, Colombia, Finland, India, Kenya, Portugal, South Africa, the United Kingdom and the United States. Pennington Biomedical Research Center Institutional Review Board approved the ISCOLE protocol, with Ethics Review Boards at each site approving local protocols. Written informed consent was obtained from parents or legal guardians, and child assent was obtained as required by local Ethical Review Boards before participation in the study. All sites adhered to a standardized protocol with all study personnel undergoing rigorous training and certification before and during data collection. Data were collected between September 2011 and December 2013. A detailed description of the sample size estimation and sampling strategy, the study design and methods used for data collection can be found elsewhere [23]. Recruitment targeted a sex-balanced sample of at least 500 children aged between 9 and 11 years from each site; a sample was deliberately stratified by SES at each site to maximize variability (rather than having nationally representative samples). A participant flow chart with the number of participants recruited and excluded at each stage is shown in Fig. 1.

### Breakfast frequency

As in our previous study [3] and a previous multi-national study [21], breakfast frequency was assessed via questionnaire by asking child participants: “How often do you usually have breakfast (more than a glass of milk or fruit juice)?” Response categories were “never” to “five days” for weekdays, and “never” to “two days” for weekend days. Weekly breakfast frequency (0 to 7 days/week) was calculated as the sum of weekday and weekend day breakfast frequency. As there is no universally accepted definition of breakfast frequency used in the literature [8–15], we employed two different definitions:

1. A 3-category definition: weekly breakfast frequency was recoded to make clear comparisons among rare (breakfast 0 to 2 days/week), occasional (breakfast on 3 to 5 days/week) and frequent (breakfast on 6 to 7 days/week) breakfast consumers.
2. A 2-category definition: weekly breakfast frequency was recoded as less than daily (breakfast 0 to 6 days/ week) or daily (breakfast on 7 days/week).

In line with our previous research [3], and to differentiate between the effects of rare, occasional and frequent consumption, the 3-category definition was the primary variable used in our analyses. The 2-category definition of “less than daily” and “daily” consumption was included to enable direct comparisons of our data with a previous multi-national study [21].

### Physical activity and sedentary time

Physical activity and sedentary time were objectively assessed using 24-h, waist-worn accelerometry across seven days; full details have been published elsewhere [23]. An Actigraph GT3X+ accelerometer (ActiGraph LLC, Pensacola, FL, USA) was worn at the waist on an elasticized belt at the right mid-axillary line. Participants were encouraged to wear the accelerometer 24 h per day for at least 7 days, including 2 weekend days (removing only for water-related activities). The minimal amount of accelerometer data that was considered acceptable was 4 days with at least 10 h of awake wear time per day, including at least one weekend day. Non-wear time and sleep time were determined [24, 25]. For waking hours, time spent sedentary, and in light PA, moderate PA, and vigorous PA were calculated using the Evenson cut-offs [26] for the following time segments: total daily, morning (wake to < 12:00 h) and afternoon (≥12:00 h to bed time). In addition to absolute min/day, sensitivity analyses were conducted with the morning and afternoon accelerometer data expressed as a proportion (%) of time to account for individual differences in wake and sleep times that affected the duration of the morning (wake to < 12:00 h) and afternoon (≥12:00 h to bed time).

### Covariates

Standing height was measured to the nearest 0.1 cm with the participant standing without shoes, with their head in the Frankfort Plane and at the end of a deep inhalation using a Seca 213 portable stadiometer (Hamburg, Germany). Body weight was measured to the nearest 0.1 kg using a portable Tanita SC-240 Body Composition Analyzer (Arlington Heights, IL). Body mass index (BMI; body mass (kg)/height (m^2^)) and BMI z-score were calculated [27]. Age, sex and the highest level of parental education were determined using demographic questionnaires completed by each participant’s parent/guardian [23]. Response categories for level of parental education were: less than high school, some high school, completed high school, some college degree, Bachelor’s degree or post-graduate degree (Master’s or PhD). Highest level of parental education was recoded into three categories: did not complete high school (low), completed high school or some college (medium), and completed Bachelor’s or postgraduate degree (high).

### Statistical analyses

SAS 9.1 (SAS Institute, Inc., Cary, NC) was used for statistical analyses. The descriptive characteristics of the study population and frequencies of breakfast consumption (using the two definitions) were calculated for each site. To address the primary aim of this study, multilevel models (SAS PROC MIXED) were computed to examine associations between breakfast frequency (independent variable) and PA and sedentary time (dependent variables). The use of multilevel models controlled for the hierarchical nature of the data. Study sites were considered to have fixed effects, and schools nested within study sites were viewed as having random effects. The denominator degrees of freedom for statistical tests pertaining to fixed effects were calculated using the Kenward and Roger approximation [28]. Age, sex, highest parental education, BMI z-score, and time-segment-specific waking accelerometer wear time were included in the models as covariates. When there was a main effect of breakfast frequency using the 3-category definition, a Bonferroni correction was applied to statistically compare differences between the three breakfast categories. To address the secondary aim of the study, potential differences in the direction of associations between breakfast frequency with PA and sedentary time across sites were examined using interaction terms in the multilevel models; site-by-breakfast frequency interactions were retained when *p* ≤ 0.05. All analyses were performed with the two different definitions of breakfast frequency. The level of statistical significance was set at *p* ≤ 0.05.

## Results

The descriptive characteristics of the participants and the percentage of participants within each breakfast frequency category stratified by site are presented in Table 1. The marginal mean minutes of PA and sedentary time by breakfast frequency using the 3-category definition are presented in Table 2. The fraction of the total variance in sedentary time and PA ranged from 7.4% for afternoon sedentary time to 25% for morning moderate PA at the school level, and ranged from 8.2% for total daily vigorous PA to 21% for afternoon sedentary time at the site level. As 86% of the covariate-dependent variable associations were significant and 100% of the covariates were significantly associated with total daily time spent in each PA intensity and sedentary time, all specified covariates were included for all analyses. The only covariates that were not significantly associated with the dependent variables were: BMI z-score for morning min/day and % time spent sedentary; parental education for morning min/day and % time in light PA; sex and BMI z-score for afternoon min/day in light PA; sex, BMI z-score and afternoon wear time for afternoon % time in light PA; age for morning min/day and % time in MPA; age for morning min/day and % time spent in VPA; age and morning wear time for morning % time in MPA; age for morning min/day and % time in MVPA.

**Table 1:** Descriptive characteristics of the sample stratified by site

Country (site)	*n* (% female)	Age (y)	BMI z-score	Highest level of parental education (%)^*a*^			3-category breakfast definition (%)^*a*^			2-category breakfast definition (%)^*a*^		Time spent in each PA intensity (min/day)				
				High	Medium	Low	Rare	Occasional	Frequent	<Daily	Daily	Sedentary	Light	Moderate	Vigorous	MVPA
Australia (Adelaide)	477 (54)	10.3 (0.5)	0.59 (1.12)	11.3	46.8	41.9	6.1	12.6	81.3	26.0	74.0	477 (60)	310 (48)	43 (13)	23 (12)	65 (23)
Brazil (Sao Paulo)	448 (51)	10.0 (0.5)	0.88 (1.43)	23.9	53.8	22.3	16.1	24.1	59.8	50.5	49.6	501 (68)	338 (53)	42 (17)	18 (11)	59 (26)
Canada (Ottawa)	516 (59)	10.0 (0.4)	0.41 (1.20)	1.9	26.4	71.7	3.1	10.1	86.8	21.5	78.5	512 (63)	304 (45)	42 (12)	17 (9)	59 (19)
China (Tianjin)	495 (48)	9.4 (0.5)	0.72 (1.53)	33.3	44.9	21.8	4.0	13.5	82.4	28.3	71.7	565 (68)	293 (54)	33 (11)	13 (7)	45 (16)
Colombia (Bogotá)	855 (51)	10.0 (0.6)	0.20 (1.04)	30.6	51.4	18.0	1.6	2.6	95.8	5.6	94.4	500 (67)	333 (49)	50 (17)	18 (10)	68 (25)
Finland (Helsinki, Espoo and Vantaa)	470 (54)	10.0 (0.4)	0.26 (1.05)	2.8	55.1	42.1	1.5	10.4	88.1	18.7	81.3	529 (67)	293 (44)	48 (15)	23 (14)	71 (26)
India (Bangalore)	546 (54)	10.0 (0.6)	0.22 (1.37)	5.1	21.8	73.1	14.5	15.9	69.6	37.9	62.1	517 (66)	340 (51)	36 (14)	13 (8)	49 (21)
Kenya (Nairobi)	501 (54)	9.8 (0.7)	0.01 (1.22)	14.4	45.9	39.7	4.2	17.0	78.8	27.2	72.9	495 (66)	330 (52)	49 (20)	23 (14)	72 (31)
Portugal (Porto)	619 (57)	10.0 (0.3)	0.86 (1.15)	45.2	33.9	20.8	2.4	3.6	94.0	14.2	85.8	553 (61)	302 (50)	39 (13)	17 (10)	56 (22)
South Africa (Cape Town)	398 (60)	9.8 (0.7)	0.33 (1.29)	47.0	38.9	14.1	11.8	22.6	65.6	49.5	50.5	489 (66)	322 (53)	46 (17)	18 (10)	64 (25)
U.K. (Bath and North East Somerset)	429 (56)	10.4 (0.5)	0.39 (1.09)	3.0	50.4	46.6	5.1	14.0	80.9	28.7	71.3	495 (59)	286 (46)	43 (13)	21 (12)	64 (22)
U.S. (Baton Rouge)	474 (59)	9.5 (0.6)	0.72 (1.28)	6.1	41.8	52.1	9.9	17.3	72.8	41.8	58.2	521 (61)	314 (51)	35 (11)	15 (9)	50 (19)
All sites	6228 (55)	9.9 (0.6)	0.45 (1.26)	19.6	42.5	37.9	6.3	12.6	81.2	27.1	72.9	513 (69)	315 (53)	43 (16)	18 (11)	60 (25)

Values are mean (SD) unless stated otherwise. Abbreviations: *BMI* Body Mass Index, *PA* physical activity; *MVPA* moderate-to-vigorous physical activity, *U.K.* United Kingdom, *U.S.* United States

^a^Values are frequencies (%) for categorical variables

**Table 2:** Associations of breakfast frequency defined using three categories with physical activity and sedentary time in children from 12 countries

		Marginal means (95% CI)			*p* for breakfast frequency main effect	*p* for breakfast frequency by study site interaction
		Rare (*n* = 389)	Occasional (*n* = 784)	Frequent (*n* = 5055)		
Sedentary	Total (min/d)	517 (510 to 524)	509 (504 to 515)	510 (507 to 513)	0.14	0.32
	Morning (min/d)	183 (181 to 186)	180 (178 to 182)	180 (179 to 181)^a^	**0.04**	0.51
	Morning (% time)	61 (60 to 62)	60 (59 to 60.5)	60 (59 to 60)^a^	**0.01**	0.27
	Afternoon (min/d)	334 (328 to 339)	329 (325 to 333)	330 (328 to 332)	0.32	0.40
	Afternoon (% time)	57 (56 to 57)	56 (55 to 56)	56 (56 to 56)	0.42	0.46
Light PA	Total (min/d)	311 (305 to 317)	318 (314 to 322)	316 (314 to 319)	0.12	0.56
	Morning (min/d)	100 (97 to 102)	102 (101 to 104)	102 (101 to 103)	0.09	0.84
	Morning (% time)	33 (33 to 34)	34 (34 to 35)	34 (34 to 35)^a^	**0.04**	0.66
	Afternoon (min/d)	211 (207 to 215)	216 (213 to 218)	214 (212 to 216)	0.17	0.54
	Afternoon (% time)	36 (35 to 37)	37 (36 to 37)	36 (36 to 37)	0.24	0.61
Moderate PA	Total (min/d)	42 (41 to 44)	43 (41 to 44)	44 (43 to 45)	0.14	0.47
	Morning (min/d)	12 (12 to 13)	13 (12 to 13)	13 (12 to 13)	**0.05**	0.26
	Morning (% time)	4.1 (3.9 to 4.3)	4.2 (4.0 to 4.3)	4.3 (4.2 to 4.4)	**0.04**	0.17
	Afternoon (min/d)	30 (29 to 31)	30 (29 to 31)	31 (30 to 31)	0.46	0.52
	Afternoon (% time)	5.2 (5.0 to 5.4)	5.2 (5.0 to 5.3)	5.2 (5.1 to 5.4)	0.57	0.58
Vigorous PA	Total (min/d)	18 (17 to 19)	18 (18 to 19)	19 (18 to 19)	0.50	0.68
	Morning (min/d)	4.8 (4.3 to 5.2)	5.2 (4.9 to 5.5)	5.2 (5.0 to 5.4)	0.12	0.25
	Morning (% time)	1.6 (1.4 to 1.7)	1.7 (1.6 to 1.8)	1.7 (1.6 to 1.8)	0.09	0.22
	Afternoon (min/d)	13 (12 to 14)	13 (13 to 14)	14 (13 to 14)	0.83	0.89
	Afternoon (% time)	2.3 (2.1 to 2.5)	2.3 (2.2 to 2.4)	2.3 (2.3 to 2.4)	0.85	0.89
MVPA	Total (min/d)	61 (58 to 63)	61 (59 to 63)	62 (61 to 64)	0.21	0.42
	Morning (min/d)	17 (16 to 18)	18 (17 to 19)	18 (18 to 19)	**0.05**	0.19
	Morning (% time)	5.7 (5.3 to 6.0)	5.9 (5.6 to 6.1)	6.0 (5.8 to 6.2)^a^	**0.03**	0.11
	Afternoon (min/d)	44 (41 to 46)	44 (42 to 45)	44 (43 to 45)	0.58	0.60
	Afternoon (% time)	7.5 (7.1 to 7.8)	7.5 (7.2 to 7.7)	7.6 (7.4 to 7.7)	0.66	0.64

Abbreviations: *95% CI* 95% confidence intervals, *PA* physical activity; rare = breakfast consumed 0–2 days/week; occasional = breakfast consumed 3–5 days/week; frequent = breakfast consumed 5–7 days/week; *PA* physical activity, *MVPA* moderate-to-vigorous physical activity

Marginal means are adjusted for age, sex, highest level of parental education, time segment-specific accelerometer and BMI z-score

Significance accepted at *p* ≤ 0.05 and shown in bold. ^a^Significantly different from rare breakfast consumption, Bonferroni adjusted

### Associations of breakfast frequency with PA and sedentary time

**Fig. 2:**
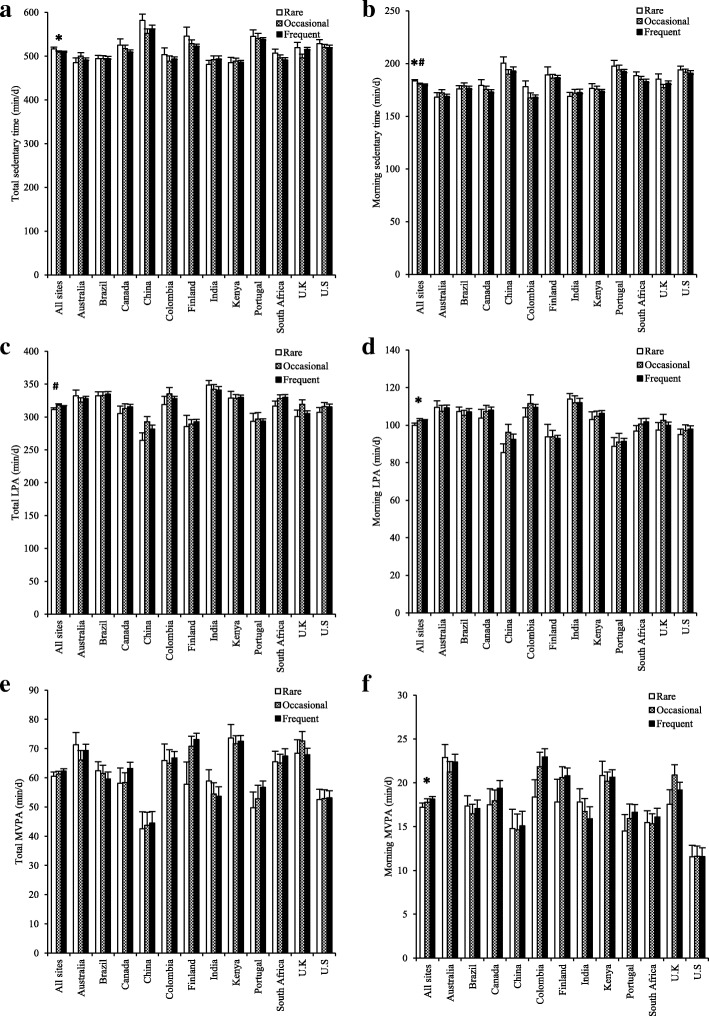
Total and morning time spent sedentary (**a** and **b**), in light physical activity (PA) (**c** and **d**) and in moderate- to vigorous-intensity physical activity (MVPA) (**e** and **f**) in rare (breakfast on 0–2 days per week), occasional, (breakfast on 3–5 days per week) and frequent (breakfast on 6–7 days per week) breakfast consumers stratified by site. Values are least squares means (error bars indicate the standard error of the mean) adjusted for age, sex, highest level of parental education, body mass index z-score and accelerometer wear time. *Significant main effect of breakfast (*p* ⩽ 0.05); ^#^significant difference between rare and frequent after Bonferonni adjustment (*p* ⩽ 0.02). No interactions with study site were found (*p* > 0.05)

Figure 2 shows PA and sedentary time for the rare, occasional and frequent breakfast consumers stratified by study site. Using this 3-category breakfast definition, breakfast frequency was not significantly associated with PA or sedentary time for the total day or for the afternoon (see Table 2). For the morning, there was a main effect of breakfast frequency on MVPA (min/day and % of morning time), moderate PA (min/day and % of morning time), light PA (% of morning time) and sedentary time (min/day and % of morning time). After adjusting for multiple comparisons, frequent breakfast consumption was associated with higher MVPA (0.3% of morning time), higher light PA (1.0% of morning time) and lower sedentary time (3.4 min/day and 1.3% of morning time) in the morning when compared with rare breakfast consumption (see Table 2). All breakfast frequency main effects were non-significant when using the 2-category breakfast definition (see Additional file 1).

### Interactions with study site

Interactions with study site were all non-significant, other than when using the 2-category breakfast definition for % morning time sedentary and % morning time in light PA (see Additional file 1). When separating the results by site, % morning time sedentary was 1.5–1.8% lower for daily breakfast consumption in Brazil (*p* = 0.02) and Canada (*p* = 0.01), but was 1.5% higher for daily breakfast consumption in India (*p* = 0.01) when compared with less than daily breakfast consumption; % morning time in light PA was 1.3–1.6% higher for daily breakfast consumption in Brazil (*p* < 0.005) and Canada (*p* = 0.03), but was 1.0% lower for daily breakfast consumption in India (*p* = 0.05) when compared with less than daily breakfast consumption. For all other study sites, associations between breakfast frequency using the 2-category breakfast definition and % morning time sedentary and % morning time in light PA were not significant.

## Discussion

In this multi-national sample of children, frequent breakfast consumption was associated with higher MVPA and light PA and lower sedentary time in the morning when compared with rare breakfast consumption, whereas no associations were found for the afternoon or whole day. The reported associations were consistent across samples of children from study sites that varied in terms of geographic, cultural, and human development. The small magnitude of these associations (reduction of ~ 3.4 min spent sedentary replaced with a mixture of light PA and MVPA each morning), may, however, have limited clinical significance.

The finding that breakfast frequency was associated with PA and sedentary time during the morning and not the afternoon is consistent with previous reports of associations during the morning period only for overall PA (accelerometer counts/min) in girls [12] and for sedentary time in boys on weekends [14]. Although the majority of empirical evidence suggests that the relationship between breakfast consumption and PA weakens as the day progresses [12, 16–18], our findings are not consistent with reports of associations with total daily MVPA in overweight Latina and African American 8–17 year old girls [13] and total daily weekend MVPA in 14–15 year old boys and girls [15]. Possible reasons for these variable results include the participant characteristics (e.g. age, adiposity status) and factors such as breakfast quality; for example, whilst breakfast quality was not associated with PA outcomes in girls, boys who consumed a poor-quality breakfast spent ~ 7 min/day more time in MVPA during weekday afternoons and evenings and boys who consumed a good-quality breakfast spent ~ 3 min/day more in MVPA during the morning on weekend days when compared with boys who did not consume breakfast [14]. It is not possible to compare our results for light PA with the pertinent literature, which has neglected this PA intensity despite evidence that changes in PA in response to breakfast consumption versus omission may be specific to the replacement of sedentary time with light PA in adolescent girls [29] and adults [16]. Thus, further research is required to establish the link between breakfast frequency, PA and sedentary time in children, controlling for factors such as breakfast quality and including light PA as an outcome.

Although it is not possible to infer causation from our cross-sectional study, the findings that associations were found in the morning and not the afternoon support a possible direct link between breakfast and PA that may result from increased exogenous glucose availability and perceptions of increased energy with reduced lethargy and fatigue in the morning [2, 16, 30]. Indeed, some [16–18], but not all [30], randomised controlled trials in adults have shown that breakfast consumption increases PA in the morning specifically. Alternatively, intentions to participate in PA, particularly in the morning, could drive breakfast consumption, in anticipation. The possibility of physiological factors (e.g., exogenous glucose availability, circadian rhythm) underpinning the link between breakfast and PA might also explain why the results were consistent across most of the 12 ISCOLE study sites that, otherwise, differed substantially in sociocultural variability. In contrast, associations between breakfast frequency and adiposity indicators differed across the ISCOLE sites [3] perhaps due to between-site geographical and sociocultural differences that could affect daily energy intake and not PA (e.g., reasons for skipping breakfast including lack of food availability or volitional weight loss in low-income versus high-income countries, or government programs for low income populations like in Bogotá).

The consistency of the reported associations across study sites suggests that our findings apply on a global level. To allow for direct comparisons with the most relevant multi-national study to date and the wider literature [21], we employed an additional ‘2-category’ definition of breakfast frequency that distinguished between ‘daily’ and ‘less than daily’ breakfast consumption. As with our previous study that examined associations between breakfast frequency and adiposity indicators [3], this 2-category definition lacked the sensitivity to detect significant associations in the present study. Further, associations were only found when comparing the extremes of rare and frequent consumption using the 3-category definition. It is possible that the use of self-report PA questionnaires and not controlling for adiposity explained the differing findings in 11 to 15 year olds from Europe, the U. S, Canada, and Israel, where daily breakfast consumption was associated with higher total daily MVPA than less than daily breakfast consumption in 34 of the 41 countries [21]. Similar to our findings, another multi-national study reported that breakfast frequency was generally not associated with objectively-measured or self-reported sedentary time or PA in 12.5–17.5 year-olds from nine European countries, although direct comparisons with our study are not appropriate because the morning and afternoon periods were not examined separately [22].

The reduction in sedentary time of ~ 3.4 min/day in the morning replaced with a mixture of light PA and MVPA reported here may not be considered by some as clinically meaningful from a public health perspective. Cross-sectional research indicates that 10 min of MVPA is associated with favourable metabolic health markers in children [31]. We found a difference of ~ 1–2 min/d higher MVPA, which falls well below this threshold, but may help to achieve it. It is difficult to ascertain how much of a reduction in sedentary time and an increase in light PA is required to achieve clinical significance due to the limited evidence base available on light PA and the lack of consensus on whether sedentary time is an independent predictor of cardiometabolic health in children [31, 32]. The magnitude of the differences in sedentary time and PA reported here are smaller than previous reports of up to ~ 5–6 min/day less time sedentary with ~ 3 min/day more MVPA for mornings and ~ 10–20.5 min/day higher MVPA across the day in frequent breakfast consumers [14, 15], and is surprising given that children who consume breakfast frequently have lower levels of adiposity, yet do not consume less energy in total during the day [6, 7]. Overall, our findings suggest that PA may not be a primary factor contributing to lower adiposity and chronic disease risk factors reported in children who consume breakfast frequently [1–3]; rather, factors related to food choices [6] and the metabolic effects of breakfast consumption (e.g., reduced glucose variability) may play an important role [16]. That said, PA promotion interventions typically target a variety of factors that are linked with PA, such as education and the environment [33], rather than focusing on a single component (i.e., breakfast). Thus, increased breakfast frequency may be a valuable addition to increase the effectiveness of such programmes, particularly for PA performed in the morning.

The limitations of our study include the cross-sectional design, which does not allow us to infer the direction of causality. Although our questionnaire used to assess breakfast frequency provided some consistency of what constitutes ‘breakfast’ between the participants (i.e., “more than a glass of milk or fruit juice”) and was in line with a previous multi-national study in children [21], the validity and reliability of this questionnaire is not known and we did not assess the quality or quantity of breakfast, which may interact with PA and sedentary time [14]. Additionally, it is possible that variations in the validity of the questionnaire existed across the study sites; indeed, typical breakfast food and drink items differ between countries [6, 8, 14, 34, 35]. It is also important to note that the association between breakfast and PA may differ by sex and between weekdays and weekend days [12, 14, 15]. We deemed that separating boys and girls and week- and weekend days was not appropriate in the present study because the focus was to examine possible differences between the study sites and definitions of breakfast consumption; stratifying the sample any further would have resulted in very low numbers in some of the breakfast categories. This approach would also require additional week- and weekend day- specific definitions of breakfast frequency and criteria for valid numbers of accelerometer days. Finally, the exclusion of participants with missing data may have resulted in some bias in our sample towards children and parents/guardians who were more compliant with study procedures.

## Conclusions

In conclusion, across 12 sites varying in geographic region and sociocultural backgrounds, frequent breakfast consumption was associated with higher MVPA and light PA and lower sedentary time in the morning when compared with rare breakfast consumption, whereas no associations were found for the afternoon or whole day. However, the small magnitude of the reported associations (i.e., a reduction of ~ 3.4 min spent sedentary replaced with a mixture of light PA and MVPA each morning) questions their clinical significance. Given the lack of consensus on the link between breakfast frequency, sedentary time and PA, the clinical significance and causal nature of the reported associations require further study in globally representative samples of children.

## Additional file

Additional file 1: **Table S1**. Associations of breakfast frequency defined using two categories with physical activity and sedentary time in children from 12 countries.’ Table showing associations of breakfast frequency defined using two categories with physical activity and sedentary time in children from 12 countries. (DOC 121 kb)

## Abbreviations

BMI: body mass index
ISCOLE: The International Study of Childhood Obesity, Lifestyle, and the Environment
MVPA: moderate-to-vigorous intensity physical activity
PA: physical activity
SES: socioeconomic status
U.K.: United Kingdom
U.S.: United States

## References

[1] Donin AS, Nightingale CM, Owen CG, Rudnicka AR, Perkin MR, Jebb SA (2014). Regular breakfast consumption and type 2 diabetes risk markers in 9- to 10-year-old children in the child heart and health study in England (CHASE): a cross-sectional analysis. PLoS Med.

[2] Smith KJ, Gall SL, McNaughton SA, Blizzard L, Dwyer T, Venn AJ (2010). Skipping breakfast: longitudinal associations with cardiometabolic risk factors in the childhood determinants of adult health study. Am J Clin Nutr.

[3] Zakrzewski JK, Gillison FB, Cumming S, Church TS, Katzmarzyk PT, Broyles ST, ISCOLE Research Group (2015). Associations between breakfast frequency and adiposity indicators in children from 12 countries. Int J Obes Suppl.

[4] Lazzeri G, Ahluwalia N, Niclasen B, Pammolli A, Vereecken C, Rasmussen M (2016). Trends from 2002 to 2010 in daily breakfast consumption and its socio-demographic correlates in adolescents across 31 countries participating in the HBSC study. PLoS One.

[5] Brown AW, Bohan Brown MM, Allison DB (2013). Belief beyond the evidence: using the proposed effect of breakfast on obesity to show 2 practices that distort scientific evidence. Am J Clin Nutr.

[6] Deshmukh-Taskar PR, Nicklas TA, O'Neil CE, Keast DR, Radcliffe JD, Cho S (2010). The relationship of breakfast skipping and type of breakfast consumption with nutrient intake and weight status in children and adolescents: the National Health and nutrition examination survey 1999-2006. J Am Diet Assoc.

[7] Timlin MT, Pereira MA, Story M, Neumark-Sztainer D (2008). Breakfast eating and weight change in a 5-year prospective analysis of adolescents: project EAT (eating among teens). Pediatrics..

[8] Arora M, Nazar GP, Gupta VK, Perry CL, Reddy KS, Stigler MH (2012). Association of breakfast intake with obesity, dietary and physical activity behavior among urban school-aged adolescents in Delhi, India: results of a cross-sectional study. BMC Public Health.

[9] Sandercock GR, Voss C, Dye L (2010). Associations between habitual school-day breakfast consumption, body mass index, physical activity and cardiorespiratory fitness in English schoolchildren. Eur J Clin Nutr.

[10] Lyerly JE, Huber LR, Warren-Findlow J, Racine EF, Dmochowski J (2014). Is breakfast skipping associated with physical activity among U.S. adolescents? A cross-sectional study of adolescents aged 12-19 years, National Health and nutrition examination survey (NHANES). Public Health Nutr.

[11] Utter J, Scragg R, Mhurchu CN, Schaaf D (2007). At-home breakfast consumption among New Zealand children: associations with body mass index and related nutrition behaviors. J Am Diet Assoc.

[12] Corder K, van Sluijs EM, Steele RM, Stephen AM, Dunn V, Bamber D (2011). Breakfast consumption and physical activity in British adolescents. Br J Nutr.

[13] Schembre SM, Wen CK, Davis JN, Shen E, Nguyen-Rodriguez ST, Belcher BR (2013). Eating breakfast more frequently is cross-sectionally associated with greater physical activity and lower levels of adiposity in overweight Latina and African American girls. Am J Clin Nutr.

[14] Vissers PA, Jones AP, Corder K, Jennings A, van Sluijs EM, Welch A (2013). Breakfast consumption and daily physical activity in 9-10-year-old British children. Public Health Nutr.

[15] Corder K, van Sluijs EM, Ridgway CL, Steele RM, Prynne CJ, Stephen AM (2014). Breakfast consumption and physical activity in adolescents: daily associations and hourly patterns. Am J Clin Nutr.

[16] Betts JA, Richardson JD, Chowdhury EA, Holman GD, Tsintzas K, Thompson D (2014). The causal role of breakfast in energy balance and health: a randomized controlled trial in lean adults. Am J Clin Nutr.

[17] Chowdhury EA, Richardson JD, Holman GD, Tsintzas K, Thompson D, Betts JA (2016). The causal role of breakfast in energy balance and health: a randomized controlled trial in obese adults. Am J Clin Nutr.

[18] Yoshimura E, Hatamoto Y, Yonekura S, Tanaka H (2017). Skipping breakfast reduces energy intake and physical activity in healthy women who are habitual breakfast eaters: a randomized crossover trial. Physiol Behav.

[19] Dialekakou KD, Vranas PB (2008). Breakfast skipping and body mass index among adolescents in Greece: whether an association exists depends on how breakfast skipping is defined. J Am Diet Assoc.

[20] Cain KL, Sallis JF, Conway TL, Van Dyck D, Calhoon L (2013). Using accelerometers in youth physical activity studies: a review of methods. J Phys Act Health.

[21] Vereecken C, Dupuy M, Rasmussen M, Kelly C, Nansel TR, Al Sabbah H (2009). Breakfast consumption and its socio-demographic and lifestyle correlates in schoolchildren in 41 countries participating in the HBSC study. Int J Public Health.

[22] Cuenca-García M, Ruiz JR, Ortega FB, Labayen I, González-Gross M, Moreno LA, HELENA study group (2014). Association of breakfast consumption with objectively measured and self-reported physical activity, sedentary time and physical fitness in European adolescents: the HELENA (healthy lifestyle in Europe by nutrition in adolescence) study. Public Health Nutr.

[23] Katzmarzyk PT, Barreira TV, Broyles ST, Champagne CM, Chaput JP, Fogelholm M (2013). The International Study of Childhood Obesity, Lifestyle and the Environment (ISCOLE): design and methods. BMC Public Health.

[24] Barreira TV, Schuna JM, Mire EF, Katzmarzyk PT, Chaput JP, Leduc G (2015). Identifying children's nocturnal sleep using 24-h waist accelerometry. Med Sci Sports Exerc.

[25] Tudor-Locke C, Barreira TV, Schuna JM, Mire EF, Katzmarzyk PT (2014). Fully automated waist-worn accelerometer algorithm for detecting children’s sleep-period time separate from 24-h physical activity or sedentary behaviors. Appl Physiol Nutr Metab.

[26] Evenson KR, Catellier DJ, Gill K, Ondrak KS, McMurray RG (2008). Calibration of two objective measures of physical activity for children. J Sports Sci.

[27] de Onis M, Onyango AW, Borghi E, Siyam A, Nishida C, Siekmann J (2007). Development of a WHO growth reference for school-aged children and adolescents. Bull World Health Organ.

[28] Kenward MG, Roger JH (1997). Small sample inference for fixed effects from restricted maximum likelihood. Biometrics..

[29] Zakrzewski-Fruer JK, Wells EK, Crawford NSG, Afeef SMO, Tolfrey K (2018). Physical activity duration but not energy expenditure differs between daily compared with intermittent breakfast consumption in adolescent girls: a randomized crossover trial. J Nutr.

[30] LeCheminant GM, LeCheminant JD, Tucker LA, Bailey BW (2017). A randomized controlled trial to study the effects of breakfast on energy intake, physical activity, and body fat in women who are nonhabitual breakfast eaters. Appetite.

[31] Ekelund U, Luan J, Sherar LB, Esliger DW, Griew P, Cooper A, International Children’s Accelerometry database (ICAD) collaborators (2012). Moderate to vigorous physical activity and sedentary time and cardiometabolic risk factors in children and adolescents. JAMA..

[32] Carson V, Hunter S, Kuzik N, Gray CE, Poitras VJ, Chaput JP (2016). Systematic review of sedentary behaviour and health indicators in school-aged children and youth: an update. Appl Physiol Nutr Metab.

[33] Speake H, Copeland RJ, Till SH, Breckon JD, Haake S, Hart O (2016). Embedding physical activity in the heart of the NHS: the need for a whole-system approach. Sports Med.

[34] Hallström L, Vereecken CA, Labayen I, Ruiz JR, Le Donne C, García MC (2012). Breakfast habits among European adolescents and their association with sociodemographic factors: the HELENA (healthy lifestyle in Europe by nutrition in adolescence) study. Public Health Nutr.

[35] O'Neil CE, Byrd-Bredbenner C, Hayes D, Jana L, Klinger SE, Stephenson-Martin S (2014). The role of breakfast in health: definition and criteria for a quality breakfast. J Acad Nutr Diet.

