# Supplementary material for: Association between breakfast frequency and physical activity and sedentary time: a cross-sectional study in children from 12 countries
Source: BMC Public Health. 2019 Feb 21;19:222. doi: 10.1186/s12889-019-6542-6 (PMC6385453; doi:10.1186/s12889-019-6542-6)
Supplement: Supplementary file 1 — Table S1. Associations of breakfast frequency defined using two categories with physical activity and sedentary time in children from 12 countries.’ Table showing associations of breakfast frequency defined using two categories with physical activity and sedentary time in children from 12 countries. (DOC 121 kb) [file 12889_2019_6542_MOESM1_ESM.doc]

**Additional file 1.**

**Table 1.** Associations of breakfast frequency defined using two categories with physical activity and sedentary time in children from 12 countries.

|  |  | Marginal means (95% CI) | | *p* for breakfast frequency main effect | *p* for breakfast frequency by study site interaction |
| --- | --- | --- | --- | --- | --- |
|  |  | Less than daily | Daily |  |  |
| Sedentary | Total (min/d) | 511 (507 to 515) | 510 (507 to 513) | 0.55 | 0.47 |
|  | Morning (min/d) | 181 (179 to 182) | 180 (179 to 181) | 0.19 | 0.17 |
|  | Morning (% time) | 60 (59 to 61) | 60 (59 to 60) | 0.17 | **0.04** |
|  | Afternoon (min/d) | 330 (328 to 333) | 330 (328 to 332) | 0.88 | 0.81 |
|  | Afternoon (% time) | 56 (56 to 57) | 56 (56 to 56) | 0.96 | 0.82 |
|  |  |  |  |  |  |
| Light PA | Total (min/d) | 316 (313 to 319) | 317 (314 to 319) | 0.71 | 0.46 |
|  | Morning (min/d) | 102 (101 to 103) | 103 (101 to 104) | 0.26 | 0.26 |
|  | Morning (% time) | 34 (34 to 35) | 34 (34 to 35) | 0.23 | **0.04** |
|  | Afternoon (min/d) | 214 (212 to 216) | 214 (212 to 216) | 0.99 | 0.71 |
|  | Afternoon (% time) | 36 (36 to 37) | 36 (36 to 37) | 0.97 | 0.79 |
|  |  |  |  |  |  |
| Moderate PA | Total (min/d) | 43 (42 to 44) | 44 (43 to 45) | 0.55 | 0.21 |
|  | Morning (min/d) | 13 (12 to 13) | 13 (13 to 13) | 0.20 | 0.23 |
|  | Morning (% time) | 4.2 (4.1 to 4.4) | 4.3 (4.2 to 4.4) | 0.25 | 0.16 |
|  | Afternoon (min/d) | 31 (30 to 31) | 31 (30 to 31) | 1.00 | 0.37 |
|  | Afternoon (% time) | 5.3 (5.1 to 5.4) | 5.2 (5.1 to 5.4) | 0.83 | 0.30 |
|  |  |  |  |  |  |
| Vigorous PA | Total (min/d) | 19 (18 to 19) | 19 (18 to 19) | 0.43 | 0.60 |
|  | Morning (min/d) | 5.2 (4.9 to 5.4) | 5.2 (5.0 to 5.5) | 0.69 | 0.48 |
|  | Morning (% time) | 1.7 (1.6 to 1.8) | 1.7 (1.6 to 1.8) | 0.67 | 0.42 |
|  | Afternoon (min/d) | 13 (13 to 14) | 14 (13 to 14) | 0.50 | 0.62 |
|  | Afternoon (% time) | 2.3 (2.2 to 2.4) | 2.3 (2.3 to 2.4) | 0.51 | 0.56 |
|  |  |  |  |  |  |
| MVPA | Total (min/d) | 62 (60 to 63.5) | 62 (61 to 64) | 0.47 | 0.28 |
|  | Morning (min/d) | 18 (17 to 19) | 18 (18 to 19) | 0.31 | 0.36 |
|  | Morning (% time) | 5.9 (5.7 to 6.1) | 6.0 (5.8 to 6.2) | 0.34 | 0.24 |
|  | Afternoon (min/d) | 44 (43 to 45) | 44 (43 to 45) | 0.77 | 0.42 |
|  | Afternoon (% time) | 7.6 (7.3 to 7.8) | 7.6 (7.4 to 7.7) | 0.87 | 0.36 |

Abbreviations: 95% CI = 95% confidence intervals; PA = physical activity; less than daily = breakfast consumed 0-6 days/week ; daily = breakfast consumed 7 days/week; PA = physical activity; MVPA = moderate-to-vigorous physical activity.

Marginal means are adjusted for age, sex, highest level of parental education, time segment-specific accelerometer and BMI z-score.

Significance accepted at *p* ≤ 0.05 and shown in bold.
